# Tape Transfer Printing of a Liquid Metal Alloy for Stretchable RF Electronics

**DOI:** 10.3390/s140916311

**Published:** 2014-09-03

**Authors:** Seung Hee Jeong, Klas Hjort, Zhigang Wu

**Affiliations:** 1 The Angstrom Laboratory, Department of Engineering Sciences, Uppsala University, Box 534, Uppsala 75121, Sweden; E-Mails: seunghee.jeong@angstrom.uu.se (S.H.J.); klas.hjort@angstrom.uu.se (K.H.); 2 State Key Laboratory of Digital Manufacturing Equipment and Technology, Huazhong University of Science and Technology, Wuhan 430074, China

**Keywords:** tape transfer printing, liquid metal alloy, microfluidic stretchable electronics, stretchable RF electronics, radio frequency identification (RFID) tag

## Abstract

In order to make conductors with large cross sections for low impedance radio frequency (RF) electronics, while still retaining high stretchability, liquid-alloy-based microfluidic stretchable electronics offers stretchable electronic systems the unique opportunity to combine various sensors on our bodies or organs with high-quality wireless communication with the external world (devices/systems), without sacrificing enhanced user comfort. This microfluidic approach, based on printed circuit board technology, allows large area processing of large cross section conductors and robust contacts, which can handle a lot of stretching between the embedded rigid active components and the surrounding system. Although it provides such benefits, further development is needed to realize its potential as a high throughput, cost-effective process technology. In this paper, tape transfer printing is proposed to supply a rapid prototyping batch process at low cost, albeit at a low resolution of 150 μm. In particular, isolated patterns can be obtained in a simple one-step process. Finally, a stretchable radio frequency identification (RFID) tag is demonstrated. The measured results show the robustness of the hybrid integrated system when the tag is stretched at 50% for 3000 cycles.

## 1. Introduction

Elastic electronics provides a new medical technology for wireless sensing and communication, with extraordinary mechanical adaptability when attached to our skin or organs. With the ultrathin integrated circuit (IC) technology and transfer process, novel applications on the skin or on other organs provide conformal contact without disturbing the user, which will significantly enhance the user experience [1–3]. However, just as in flexible or rigid systems, this IC technology has limitations in that it is expensive and that the thin films have high impedance when made in larger sizes [4]. Hence, printed circuit technology can be used to complement this technology, where modularity allows for smaller advanced systems by combining a printed circuit with a hybrid assembly of rigid active components [5]. In addition, in order to obtain low impedance in large-areal electronics, the conductors need to have a large cross section as well as to be compliant and the contacts between the rigid active components and the stretchable interconnect need to survive stretching of the circuit.

One alternative solution is to use a microfluidic approach [6], where highly conductive, liquid-alloy-based stretchable circuits are placed inside an elastic polymer encapsulation and rigid active components are assembled on the stretchable circuit by hybrid integration [7]. Independent of the size of their cross section, gallium (Ga)-indium (In)-based liquid alloys provide high compliance and the contact to rigid components will not break when they are stretched. However, this technology needs to be adapted to batch technology for rapid prototyping and potential future high throughput production. To pioneer the technology, a few demonstrations were carried out with capillary structures and manually filling the liquid alloy with a syringe [8–10]. After this, basically three different methods were demonstrated for batch processing of stretchable microfluidic circuits. Initially, batch processing was demonstrated by using a structured permanent polydimethylsiloxane (PDMS) layer as the stencil mask and a squeegee to apply the liquid alloy. Gold at the bottom of the PDMS via structures increases the wettability and reduces somewhat the risk of contamination on the top surface of the PDMS [11]. This printing technology is however somewhat cumbersome, first demanding lithographical patterning of metal on a PDMS layer and then alignment and bonding of another PDMS layer with through holes to it, before the deposition of the liquid alloy. Further, the top surface of this layer is easily contaminated.

Several studies on liquid alloy patterning have focused on direct printing techniques such as dispensing [12], 3D-printing [13], ball point writing [14] and micro contact printing [15]. Casting of liquid alloys [16] and direct laser patterning process of liquid alloys [17] for soft electronics have also been reported for liquid alloy applied device fabrication, but these techniques still have limitations in making parallel processing effective in large area electronics and allowing cost-effective high throughput processing. Recently, we proposed and verified a batch-based fabrication on a flat semi-cured PDMS substrate by using metal stencil masks [18]. However, the major disadvantage was the transfer of the stencil mask, where isolated patterns are not accepted and delicate structures are easily deformed. Most recently, masked deposition of a liquid alloy, using tin (Sn) foil and In to get a selective wetting of the Sn, was kept in a nitrogen environment chamber for at least 24 h to let the Sn react with the liquid alloy and form a new liquid alloy that wets the PDMS well [19]. This process has many difficult steps and demands that the new liquid alloy have the desired properties. Very recently, by introducing a tape transferring technique, we developed a new rapid prototyping technique to make molds in the traditional soft lithography, thereby replacing the high cost and time consuming SU-8 molds made by photolithography. This simple approach offers a reliable and fast patterning technique at reasonable resolution [20].

By combining this newly developed tape transfer technique and our previous liquid alloy printing technique, we present here one further step toward the goal of a low-cost, high throughput rapid prototyping technology for stretchable printed circuits. Tape transfer printing of a liquid metal alloy offers a simple and robust liquid alloy patterning technique that allows for isolated pattern printing with one step masking. At the same time, the limitations of this batch technology are discussed and finally a printed stretchable radio frequency identification (RFID) tag is demonstrated.

## 2. Experimental

### 2.1. Materials

The liquid alloy used, Galinstan^®^ (Geratherm Medical AG, Geschwenda, Germany) consists of Ga, In and Sn, which means it has a low melting point at −19 °C. It has high electrical conductivity, 3.46 × 10^6^ S/m at 20 °C. PDMS kit (Elastosil RT601A and B, Wacker Chemie, Munich, Germany) was used for the elastomeric polymer as a packaging material. Tapes were selected for masking (L&M series, Ritrama, Manchester, UK) as well as for tape transferring (ApliTape 4050, R Tape Corp., South Plainfield, NJ, USA), where proper adhesive strength is required. A painting roller for art painting from a local supplier was used for liquid alloy printing. A 4-inch silicon wafer was used as the supportive substrate for later processing.

### 2.2. Tape Transfer Printing

**Figure 1.**
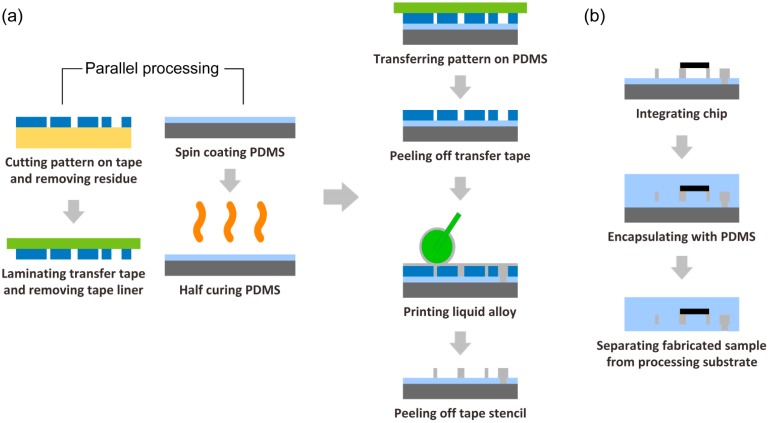
Processing steps of (**a**) tape transfer printing and (**b**) device fabrication.

The whole process consists of three major steps: tape transfer printing of liquid alloy, hybrid integration and finally encapsulation, as shown in Figure 1. PDMS was prepared by mixing base and cross-linker with 9:1 weight ratio in a disposable plastic cup. Air bubbles were first removed in a vacuum chamber and further in a refrigerator. A mask for patterning the liquid alloy was designed with AutoCAD (Autodesk, San Rafael, CA, USA) and engraved onto an adhesive tape by a mechanical cutting plotter (CraftRobo Pro CE5000-40-CRP, Graphtec Corporation, Tokyo, Japan). Cutting conditions were optimized for the tape with a dedicated knife (CB09UA-5, Graphtec Corporation). In parallel, a thin PDMS layer on a processing substrate was prepared with a spin coater (WS400B-6NPP-Lite, Laurell Technology, North Wales, PA, USA). The PDMS layer was half-cured on a hot plate. Half-curing improves the wetting of the liquid alloy on the PDMS surface when the liquid alloy is printed, and improves the bonding strength between the secondary encapsulating PDMS and the first layer of PDMS [11]. The adhesive strength of the transfer tape should be weaker than that of the tape mask to the printing substrate, so that the tape mask stays on the PDMS surface when the transfer tape is peeled off. However, the adhesive strength of the transfer tape should be strong enough to keep the cut structured patterns on the adhesive (tape) mask. The as-prepared tape mask on liner was laminated manually by a scriber or by a laminator (PL714, Peach Australia Pty Ltd., Mosman, NSW, Australia) to a transfer tape. In order to keep these cut patterns on the mask, lamination is a critical step for obtaining sufficient adhesion of the whole surface of the tape mask to the transfer tape. The liner was removed from the tape mask and unwanted parts (pattern residues) or opening parts in the tape mask were also removed manually with a needle or tweezers. Then, the adhesive mask on the transfer tape was gently transferred to the as-prepared half-cured PDMS substrate through the laminated transfer tape. After removing the transfer tape from the adhered tape mask on the PDMS, the cut tape mask was adhered on the PDMS. The liquid alloy was then printed with a sponge head roller by rolling it over several times. The printed liquid alloy patterns were made ready by slowly peeling the tape mask off the substrate.

### 2.3. Hybrid Integration Process for Device Fabrications

An ultra-high frequency (UHF) RFID chip (Higgs-3, Alien Technology, Morgan Hill, CA, USA) was mounted onto the printed liquid alloy circuit manually or by a pick-and-place machine. The liquid alloy antenna was 44 mm long and 10 mm wide, being modified from the reference antenna design (ALN-9610, Alien Technology) by the chip provider. The PDMS packaging was 1 mm thick due to the chip packaging. The electrical connection between the mounted chip and the patterned liquid alloy circuit was tested with a multimeter (34405A, Agilent Technologies, Santa Clara, CA, USA) to ensure good electrical connection. Finally, the integrated sample was encapsulated with a second layer of PDMS. Half-cured PDMS can help to achieve strong bonding strength in the encapsulation. To remove the bubbles captured in the encapsulating step in the encapsulating PDMS, as well as to obtain an even surface, the encapsulated sample was exposed to the air for a while at room temperature before it was placed in an oven (UM 400, Memmert, Germany) for the secondary layer PDMS curing and bonding between the two PDMS layers.

An isolated pattern was processed through the tape transfer printing and a simple proof-of-concept device with LEDs (SMD LED 2V, Orange, Kingbright, Germany) was fabricated using the same integration process. Copper wires to connect a power supply were mounted before the encapsulation. A power supply (QL355P, TTi Inc., Huntingdon, UK) was used to drive the sample at 2 V.

### 2.4. Observation and Characterization of the Printed Liquid Alloy Patterns

The printed liquid alloy circuits were observed under an optical microscope (Provis AX70, Olympus, Tokyo, Japan). An optical profiler (Wyko NT1100, Veeco, Plainview, NY, USA) was used to observe the surface morphology and the shape of the patterned liquid alloy circuits. The line width and spacing of the cut tape mask and the printed liquid alloy pattern were measured with a calibrated optical microscope program. The thickness profile of printed liquid alloy patterns was measured with the optical profiler.

### 2.5. Performance Evaluation of Stretchable RF Electronics

Strain and cycling tests of stretchable RF electronics, embedding liquid alloy antenna and an RFID chip, were conducted in a manual stretching frame as presented in the previous work [18]. The cycling performance of the sample was tested at around 1 Hz. The fabricated RF electronics was operated with an RFID reader (ALR-9900+, Alien Technology) connected to a reader antenna (ALR-8696-C, Alien Technology) in the frequency range of 865.7–867.5 MHz. To investigate the RFID performance, read rates were monitored during strain and cycling. Read rates from a total of 1000 reads were averaged. The distance between the RFID reader antenna and the stretchable RFID tag was 1.5 m and the angle of the sample toward the reader antenna and co-axial position of stretchable RF electronics to the RFID reader antenna were maintained during the whole test.

## 3. Results and Discussion

### 3.1. Printed Liquid Alloy Patterns

**Figure 2.**
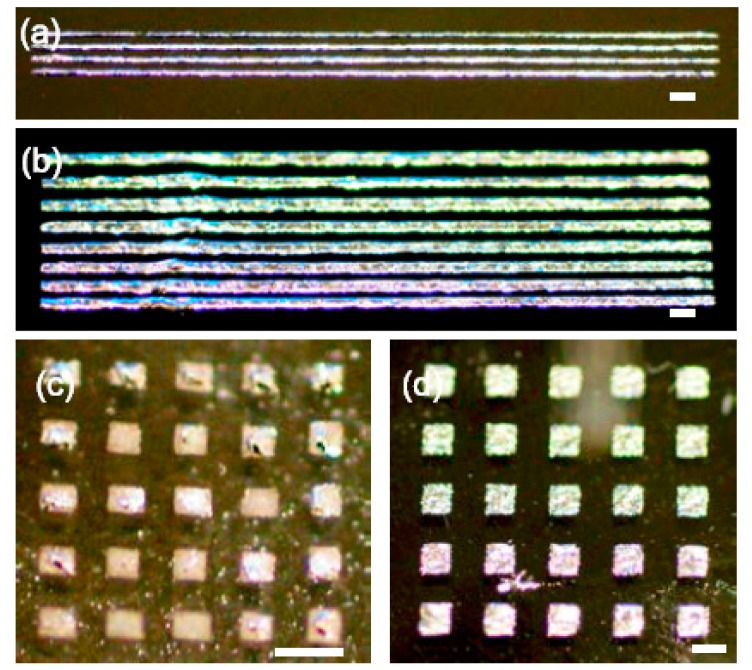
Printed patterns of liquid alloy on PDMS using tape transfer printing: (**a**) Line patterns of 200 μm width with a space of 500 μm; (**b**) Line pattern of 500 μm width with different spacing, Square dot patterns of (**c**) 500 μm; and (**d**) 1 mm. The scale bar is 1 mm.

Tape transfer printing was examined to explore the resolution and print quality of the newly developed process. The line width of the printed liquid alloy patterns and the line width of the cut mask patterns were compared with several different line widths and with different spacing between lines. The printed liquid alloy patterns via a tape mask are shown in Figure 2. The tape mask's minimum cut line width produced by the mechanical cutting plotter was 200 μm and the corresponding printed line width produced by tape transfer printing was 150 μm.

**Figure 3.**
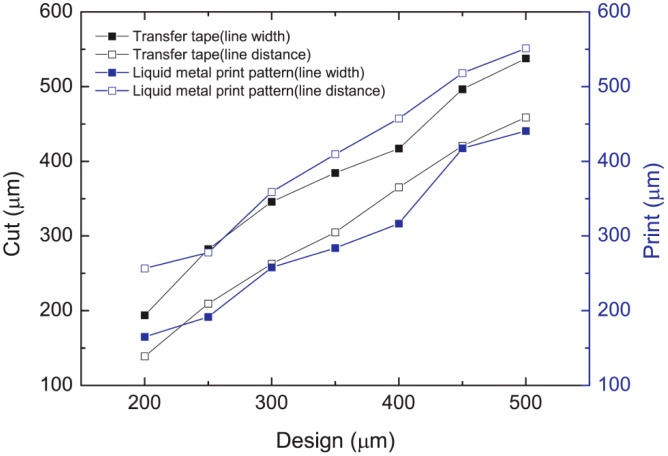
Comparisons of the line width and spacing of the mask design, the cut mask and the printed liquid alloy patterns.

**Figure 4.**
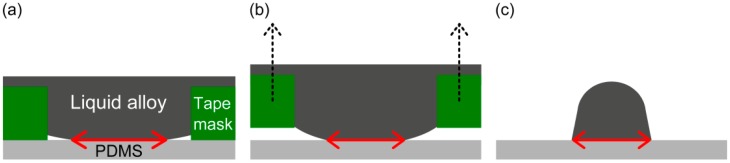
Conceptual drawing of the cross section structure of tape transfer printing of a liquid alloy where the red arrow represents wetting length of liquid alloy on PDMS (not scaled): (**a**) The printed liquid alloy on PDMS with a tape mask; (**b**) The tape mask peeling off from PDMS; and (**c**) Line width decrease of the printed liquid alloy pattern after mask removal.

The results in Figure 3 indicate that the mask design needs an additional 50–100 μm width to reach a desired printed line width. The difference between the printed patterns and the cut adhesive mask patterns may be caused by the wetting of the liquid alloy to the PDMS surface and the tape mask's sidewall, Figure 4.

The small gap at the corner between the PDMS substrate and the adhesive mask may come from the fact that the liquid alloy is not so easily pressed down at the corners using the printing roller and that the wetting is not sufficient to fill the unmasked area. If the liquid alloy does not wet the PDMS much more than to the adhesive mask sidewall, the printed liquid alloy at the corner region could be delaminated and also be deformed when the tape mask is peeled off from the PDMS surface. In both cases, the line width of the printed liquid alloy pattern would become smaller than the line width of the adhesive mask. This difference of line widths between the cut patterns and the printed patterns may also be affected by such printing conditions as the printer head surface, printing pressure and printing times. A tape mask cut by a cutting plotter has a sidewall slope originating from the knife edge shape and tape hardness, which may also cause line width variations [20]. This variation could be improved by controlling the liquid alloy wetting on the PDMS surface as well as optimizing better cutting process conditions with a higher precision mechanical cutting plotter or a laser engraver. However, this variation can be acceptable in the case of a hundred-micron scale device fabrication.

**Figure 5.**
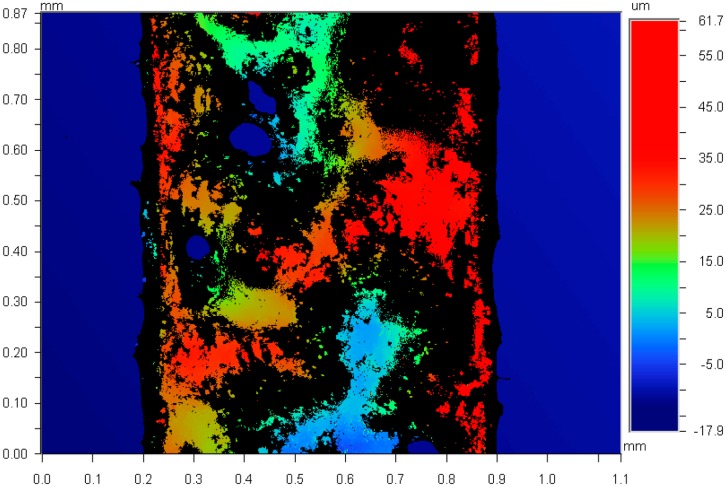
Optical profiler image of a printed liquid alloy line.

The optical profiler image in Figure 5 shows the surface topography of a printed liquid alloy pattern. It was rough and non-uniform at a thickness of the micron length scale determined by the printing roller head. The sponge surface of the roller head was porous and the liquid alloy surface seems to replicate its surface morphology. A smooth surfaced roller head may reduce the surface roughness of the printed liquid alloy patterns and also increase their uniformity. The thickness of the printed pattern of liquid alloy was in the range from 50 to 80 μm, which is similar to that of the tape mask.

Usually a gallium-based liquid alloy such as Galinstan has a thin gallium oxide layer, or so-called oxidized skin, which exists naturally in surrounding air and affects its rheology and wetting behavior significantly [9]. This oxidized skin helps the liquid alloy to keep its patterned shape after printing and removing of the tape mask. Otherwise, the liquid alloy could flow out on the PDMS surface. The surface energy of PDMS will also affect the shape of the pattern of liquid alloy. Hence, controlling the oxidized skin growth may improve the quality of the patterning process.

### 3.2. Isolated Patterns with Hybrid Integrated LED

**Figure 6.**
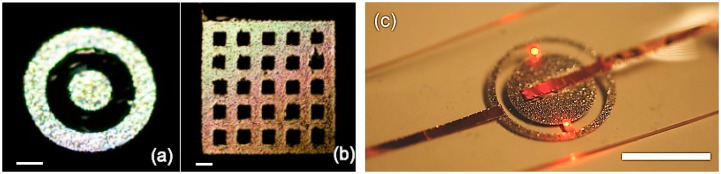
Examples of patterns from a tape mask with isolated pattern fabricated by the tape transfer process: (**a**) Co-axial circular pattern; (**b**) Rectangular mesh pattern made by a tape transferred mask that has isolated patterns; and (**c**) LED lighting device with an isolated liquid alloy conductor structure. The scale bar is 1 mm in (a, b) and 10 mm in (c).

Isolated patterns could be placed directly on a PDMS substrate using a one-step tape transfer printing. A fabricated LED lighting device with a simple isolated pattern fabricated by tape transfer printing is shown in Figure 6. Two LEDs were mounted on the isolated patterned liquid alloy contact pads and turned on by a power supply at 2 V. Compared to the recently reported solution using a metal stencil mask for liquid alloy printing [20], the newly developed tape transfer printing is much easier to handle. More importantly, it allows for simple one-step printing of isolated patterns.

Tape transfer printing for liquid alloy patterning is a new microfluidic process designed for a stretchable medium. For instance, it can pattern a long conducting line of liquid alloy on a PDMS substrate, e.g., a millimeter scale stretchable antenna which needs a long conducting pattern in a small area with high efficiency. This patterning process is parallel based, cost-effective and applicable for large areas. In particular, our tape transfer printing is attractive for rapid prototyping since it is a fast, easy, one-step process, even for an isolated pattern without any alignment of several masks. Together with hybrid integration, it could be done within one hour, due to the quick tape mask preparation via fast and precise mechanical cutting and the parallel processing concept. Further, it is compatible with other processes for large area patterning. However, its patterning resolution is limited to around 150 μm, which places constraints on the working resolution of the currently used mechanical cutting plotter and wetting imperfection of the liquid alloy during printing. Printed patterns could not present very sharp edges and controlled thickness, and the tape mask is a one-time use disposable.

In addition, this technique is a rather flexible prototyping process. For example, the mask material is not limited to the adhesive tape currently used. It could be any kind of flexible or even slightly rigid sheet material that can be engraved by a mechanical cutting plotter or a laser engraver if higher venture costs allow. An overhead transparency film could serve as a mask as well. In this case, the transparency should be mounted on the transfer tape first and then the unwanted residues should be removed after cutting. The rest of the process will be the same for the printing. This kind of approach offers researchers more freedom to control the adhesion strength between the printed substrate and mask material, since the contacted surface to the substrate can be easily surface modified to tune its surface energy and hence its adhesion strength to the targeted printing substrate. All these potential changes could significantly enhance the availability and reliability of the process in various situations, although further investigation is required. Alas, a major limitation of tape transferring printing is the final step of removing the adhesive mask. It affects the resolution and hinders further possible mass production. This might be circumvented by introducing a stiffer low surface energy masking tape or foil, or a solvent soluble one, or UV/thermal controlled adhesive, which could be more easily removed.

Finally, this is an environmentally friendly process that is possible to do in an ordinary laboratory without chemical or clean room instrumentation, and the produced waste can be easily recycled without any special treatment.

### 3.3. Performance Test of a Stretchable RFID Circuit

**Figure 7.**
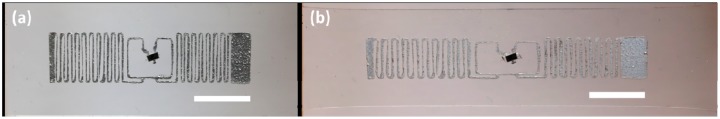
Stretchable RFID circuit with liquid alloy antenna and an integrated RFID chip: (**a**) Un-stretched; and (**b**) Stretched to 50% of the initial length. The scale bar is 10 mm.

A stretchable RFID tag was fabricated by tape transfer printing of a liquid alloy antenna circuit, followed by hybrid integration of assembling an ultra-high frequency (UHF) RFID chip on it and subsequent PDMS encapsulation, Figure 7. The liquid alloy antenna was 44 mm long and 10 mm wide, being modified from the reference antenna design (ALN-9610, Alien Technology) by the chip provider. The PDMS packaging was 1 mm thick. The performance of the rigid chip integrated stretchable RFID device was reliable and comparable to the performance of the reference RFID tag from the supplier.

A stretchable RFID tag was fabricated with a liquid alloy antenna by tape transfer printing and RFID chip integration, Figure 7. The read rates were tested with an RFID reader, Table 1. The improved read rates when being elongated might be from the change of the antenna shape, resulting an improved impedance and frequency matching, as well as in wider bandwidth. Further investigations are required to verify the exact reasons. To conclude, the performance of the rigid chip integrated stretchable RFID tag was reliable and comparable to the performance of the reference RFID tag from the supplier.

The cycled sample maintained its initial shape, such as length of the device and the PDMS packaging surface. A rigid chip in an elastomeric package can cause stress concentration around the chip and delamination of the chip from the elastomer surface. A bare die embedment, instead of a packaged chip, can minimize these problems significantly.

To summarize, microfluidic circuits with liquid alloy conductors made by tape transfer printing have the potential to become a high throughput and low-cost technology. Our vision is that this technology has the potential to be used with elastic electronics as a compliant and comfortable contact technology for our bodies for wireless sensing and communication, or for soft robotics.

## 4. Conclusions

Tape transfer printing of a liquid alloy can be successfully applied to microfluidic stretchable electronics fabrication in batch processing. Tape transfer printing of a liquid metal alloy is a simple and robust printing technique for liquid alloy microfluidic circuits, which allows for isolated pattern printing with one-step masking, although with a low resolution of around 150 μm. The measurement of the demonstrated stretchable RFID tag indicated the robustness of the hybrid integrated system using the presented technique, when it was stretched at 50% for 3000 cycles. It has the potential to provide a low-cost and high throughput rapid prototyping technology for stretchable printed circuits. Further work on a new type of easily removed masking materials or processes would be valuable for an even higher throughput and reliability for potential future mass production.

## Figures and Tables

**Table 1.** Performance of the stretchable RF electronics with strain and cycling.

**Stretchable RF Electronics**			**Reference Antenna Read Rate (Reads/s)**

**Strain Cycle (Times)**	**Read Rate (Reads/s)**		

	**Un-stretched**	**Stretched (50%)**	
0	110	159	156
1000	114	159	–
2000	137	159	–
3000	148	159	–
